# A scenario for magnonic spin-wave traps

**DOI:** 10.1038/srep12824

**Published:** 2015-08-17

**Authors:** Frederik Busse, Maria Mansurova, Benjamin Lenk, Marvin von der Ehe, Markus Münzenberg

**Affiliations:** 1 I. Physikalisches Institut, University of Göttingen, Friedrich-Hund-Platz 1, 37077 Göttingen, Germany; 2 Institut für Physik, Greifswald University, Felix-Hausdorff-Straße 6, 17489 Greifswald, Germany

## Abstract

Spatially resolved measurements of the magnetization dynamics on a thin CoFeB film induced by an intense laser pump-pulse reveal that the frequencies of resulting spin-wave modes depend strongly on the distance to the pump center. This can be attributed to a laser generated temperature profile. We determine a shift of 0.5 GHz in the spin-wave frequency due to the spatial thermal profile induced by the femtosecond pump pulse that persists for up to one nanosecond. Similar experiments are presented for a magnonic crystal composed of a CoFeB-film based antidot lattice with a Damon Eshbach mode at the Brillouin zone boundary and its consequences are discussed.

The manipulation of spin-wave frequency and propagation characteristics is of great interest for the design of switching devices such as logic gates in the field of spintronics, and the number of studies in this field grows rapidly^1^,^2^. The most promising techniques include (i) current-injected magnetic solitons in thin films with perpendicular anisotropy^3^, and (ii) a change in the ferromagnet’s temperature and therewith its saturation magnetization. The latter can either be brought about by direct contact with e.g. a Peltier element, demonstrated by Brillouin-Light-Scattering (BLS)^4^, or it can be optically induced: The authors of a recent study^5^ show that by punctually heating up a ferrimagnetic stripline by up to Δ*T* = 70 K using a focused cw laser, magnetostatic surface spin waves propagating along the stripline are trapped in the resulting potential well. In this work, we address the generation of a spin-wave trap on a magnonic crystal by means of a temperature gradient induced by intense ultrashort laser pulses.

In contrast to the experiments mentioned above, rich magnetization dynamics can be produced without any need for direct contact with the sample by using short optical pulses. One approach is using the inverse Faraday effect, which in combination with a spatially shaped pump spot can create propagating droplets of backward volume magnetostatic waves^6^. On the other hand, the technique applied in this work relies on local, short wavelength spin-wave generation by a thermally induced anisotropy field pulse to start magnetic oscillations.

The spin-wave spectrum originating from such optical excitation is usually quite broad: Ultrafast demagnetization leads to a dense population of high energy excitations which then gradually decays into lower energy spin-wave modes on a timescale of a few picoseconds^7^. The result is an overpopulation of the lowest energy states which on a continuous film are given by the uniform precession or Kittel mode and by a series of perpendicular standing spin waves. Using microstructured magnetic films (magnonic crystals) energy is as well transferred into a Damon-Eshbach type mode whose frequency can be tuned in a wide range by choosing appropriate lattice parameters^8^.

A common method to access these dynamics, described by the Landau-Lifshitz model of magnetization precession, makes use of the magneto-optical Kerr effect (MOKE)^9^ for the detection of spin waves at ultrafast timescales. Both temporal and spatial information can be obtained by applying time resolved scanning Kerr microscopy (TRSKM). Using this technique, propagating spin-wave modes have been observed by focusing pump pulses with a full width half maximum (FWHM) of only 10 μm on a thin Permalloy film^10^.

In this work, we use CoFeB as the sample material due to its low Gilbert damping (*α* = 0.006) and high saturation magnetization, resulting in a large group velocity 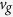 ≳ 25 km/s^11^ in an in-plane magnetized film. In order to saturate the 50 nm CoFeB sample, an external magnetic field 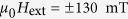 is applied at 20° to the sample plane. Due to a strong in-plane dipolar anisotropy field, the resulting magnetization will be canted 2–3° with respect to the sample plane, enabling a longitudinal MOKE detection scheme. Ultrashort laser pulses from a regeneratively amplified Ti:Sapphire system are used to (i) excite the magnetization dynamics, (ii) probe the magnetic response of the magnonic crystal, and (iii) create a spin-wave trap scenario.

## Results

**Figure 1:**
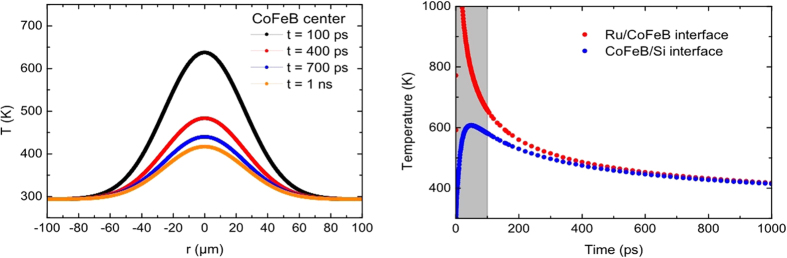
Lateral temperature distribution for different moments in time (left) and simulated time evolution of the sample’s temperature for different depths (right). Gray shadowed area in the right panel indicates the first 100 ps, when the heat gradient is rather large.

In order to find the conditions (e.g. laser pulse power) for spin-wave confinement, first the numerical simulation package *COMSOL* has been used to calculate the thermal response of a thin film to ultrafast laser excitation. The sample system for these calculations consisted of 3 nm of ruthenium capping a 50 nm cobalt-iron-boron (Co_20_Fe_60_B_20_) magnetic film on a Si(100) substrate. The results of the simulation are shown in Fig. 1: In the beginning, the laser pulse produces a sudden rise in temperature. After thermalization of optically excited electrons and equilibration of the spin and phonon subsystems, known to take place on timescales of ≈100 fs and ≈1 ps respectively, the modeling yields an effective sample temperature, i.e the temperature of the magnetic system. During the first ≈100 ps the spatial as well as the temporal heat gradient are rather large, whereas at later times the temperature remains at a high mean value and a negligible depth profile.

While the temperature is mainly homogeneous throughout the sample depth, it changes significantly across its plane, as shown in Fig. 1 (left). The Gaussian distribution of laser intensity in the pump spot produces a temperature profile that persists longer than the lifetime of the observed coherent spin-wave modes. During this time (up to 1 ns), no significant heat transport takes place on a micrometer scale and the FWHM of the lateral temperature distribution remains unchanged. In accordance with the Curie-Weiss law, the temperature increase quenches the sample’s saturation magnetization which leads to a change in the spin-wave frequency spectrum.

Experiments were performed separating the pump and probe spots on the sample and measuring the magnetization dynamics as a function of pump-probe distance, allowing us to determine the shift in magnetization oscillation frequency along the lateral temperature gradient.

**Figure 2:**
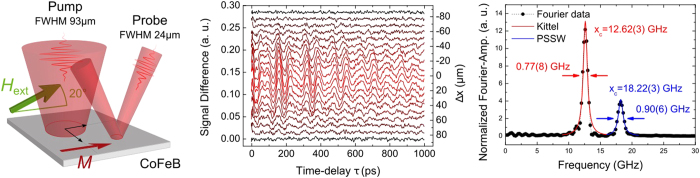
Analysis of TRMOKE data. Left: pump-probe geometry, direction of the external magnetic field 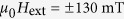 and direction of magnetization *M*. For quantitative analysis, the difference between both field directions is calculated and an incoherent background which originates from high frequency and high-*k* magnons excited by the intense pump beam^9^ is subtracted. A coherent oscillation of the magnetization is visible (center). A fast Fourier transform is performed (right). In the frequency domain, two modes are identified as the uniform precession (Kittel *k* = 0 mode) and perpendicular standing spin waves (PSSW). In order to determine the central frequency *x*_*c*_ of each mode, a Gaussian is fitted to the data.

Using a variable time delay 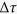 between pump and probe pulses, the time-resolved magneto-optical Kerr effect (TRMOKE) reveals magnetization precession on timescales of up to 1 ns, that changes phase by *π* when reversing magnetic field direction and the resulting signal is analyzed in frequency domain (see Fig. 2). The frequency resolution is limited by the temporal scan length and Fourier transform to 0.5 GHz minimum line width; lateral resolution is given by the probe-spot diameter with 24 *μm* FWHM.

The dataset presented in Fig. 2 has been obtained on a continuous CoFeB reference film of thickness *d* = 50 nm. Two modes of magnetic precession are observed: in-phase precession of all spins (uniform Kittel mode) at 12.6 GHz and a first order (i.e. *n* = 1) standing spin wave with wave vector *k* = *nπ* *d*^−1^ perpendicular to the sample plane (PSSW) at 18.2 GHz^8^,^9^. Both Kittel and PSSW modes have no wave vector components in the lateral direction, i.e they do not propagate on the sample but have a rather localized character at the spot of (optical) excitation. Consequently, spatially resolved measurements should show no significant precession outside of the pump laser spot.

**Figure 3:**
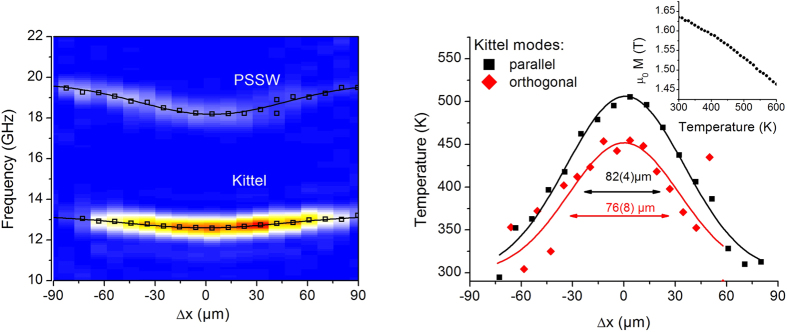
Experimental results on a continuous film. In (left) open squares represent fitted peak positions. The precession frequency observed after optical excitation is not constant across the pump spot. For the Kittel mode, a local magnetization can be calculated. Together with the magnetization curve shown in the inset, the temperature of the spin system can be derived (right). Closed diamonds correspond to a displacement of the probe with respect to the pump spot in a direction orthogonal to the applied field, squares depict a parallel displacement. Solid lines are Gaussian fits to the data. Curves are offset so that the frequency dip of the Kittel mode is centered at Δ*x* = 0.

Fig. 3 (left) shows the color-coded Fourier spectrum of magnetization oscillation as a function of spatial separation Δ*x* between the centers of pump and probe spot parallel to the external field direction. The precessional amplitude (in the color code) depends on the distance to the center of the pump pulse, due to the laser intensity profile and the localized character of the observed modes. Additionally, the frequency is strongly position dependent. This is a consequence of increased disorder caused by the intense heating, which leads to a decrease in saturation magnetization and therefore to a change of the spin-wave spectrum.

Using the dependence of Kittel mode frequency on the saturation magnetization *M*_S_


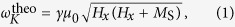


the frequency shift can be used to calculate the laser induced temperature increase for each delay. In Eq. (1) the saturation magnetization *M*_S_ is the only free parameter, such that 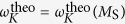, with *H*_*x*_ temperature and position independent. Comparing this with the experimentally observed frequency profile 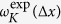 (open squares in Fig. 3, left), a corresponding profile in magnetization 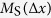 is calculated. The magnetization profile is then compared to the magnetization curve *M*(*T*) obtained for a CoFeB sample of equal thickness and composition using a Vibrating Sample Magnetometer (VSM) (Fig. 3, inset). The resulting position dependent temperature profile is shown in Fig. 3, right.

While we expect that the Kittel and PSSW do not propagate across the sample plane, in magnonic crystals composed of periodically arranged antidots the optical excitation of dipolar Damon-Eshbach surface waves (DE) of selective wave vector has been shown^8^. The wave vector of excited DE surface modes lies either perpendicular or at 45° to the external magnetic field, along the lattice vector of the magnonic crystal’s primitive cell^8^. Since the most significant density of states is expected for DE spin waves with wave vectors close to the Brillouin zone boundary where the bands are flattening^2^,^12^, only short propagation distances are expected: within a certain band width we derive group velocities of 1.3 km/s at the middle of Brillouin zone reducing further towards higher k-vector. Therefore, the propagation length estimated in the antidot lattice is reduced sigificantly up to 1.3 μm towards the zone boundary for the Bloch mode. In addition we note that the DE spin-wave dispersion is derived for continous thin films. With the lateral variation of the temperature gradient here, it is a good local approximation for wavelengths much smaller than the magnetization gradient only. The DE spin-wave dispersion is locally modified by a temperature dependent magnetization 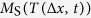 as well.

**Figure 4:**
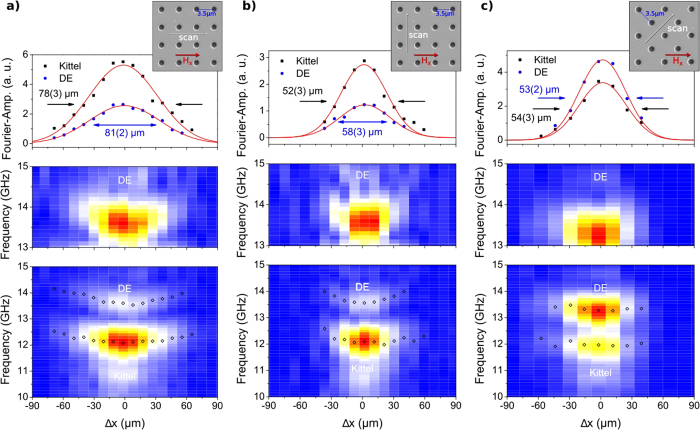
Experimental results on a magnonic crystal. Bottom: Fourier-spectrum in analogy to Fig. 3(left) with the additional magnonic Damon-Eshbach (DE) Bloch mode. Above, the DE-mode is shown with an adapted color code for better visibility. The Fourier amplitude for fundamental Kittel mode (squares) and dipolar DE mode (circles) is plotted as a function of the relative position between pump and probe. No significant spatial widening is observed for the DE mode frequency profile. Top: Scanning direction relative to the applied field and to the magnonic crystal.

Magnetization dynamics measurements on a magnonic crystal and its analysis are presented in Fig. 4 where pump and probe beam were separated (a) parallel, (b) orthogonal and (c) at 45° to the external magnetic field. An additional (magnonic) Damon-Eshbach mode is visible (bottom images of Fig. 4), corresponding to a mode at the Brillouin zone boundary with *k* = *π*/*a*. The DE mode splitting at the Brillouin zone boundary is not observed due to the limited frequency resolution of the measurements. Similarly to the Kittel and PSSW modes, the DE precession frequency shows a Gaussian dependence on position with a minimal frequency at the position of maximal pump intensity (i.e temperature), where a shift by 0.5 GHz is observed. Consequently, spin waves in the antidot lattice would have to match the frequency shift while propagating, thus requiring frequency upconversion. This is in contrast to spin-wave wells created by a finite sized magnetic structure, resulting in a discrete energy spectrum and pointing to a coherent superposition of the reflected spin-waves^13^. Due to the large dimension of the laser spot size and the short spin-wave propagation distance no coherent effects are observed in our case. As a result we observe a continously varying frequency profile along the pump spot.

The Fourier transformed spectra for each measurement are plotted in the top row of Fig. 4. Solid lines represent Gaussian fits to the experimental points, the fitted widths amount to around 50–80 μm. The sightly larger value for the horizontal measurement is due to an ellipticity of the pump focus. By comparison of the FWHM of the Kittel and DE mode, the surface mode’s propagation characteristics can be determined. Propagation of the DE mode would be visible as an asymmetric broadening with respect to the Kittel mode for the scan direction perpendicular to the applied field. In Fig. 4(a), for the case of the scan direction parallel to the applied field, both modes show the same FWHM. Also for the orthogonal and 45° configuration (Fig. 4(b,c)) the DE mode shows the same FWHM, which is somehow expected with the short propogation lenght of the DE mode and the resolution also determined by the probe spot diameter of 24 *μm* FWHM. However their intensities interchange for the 45° configuration and the DE mode becomes larger in its Fourier amplitude.

## Discussion

The presented experiments and consequences from their analysis carry out two important points: Firstly, we observe a magnetization profile 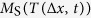 that follows the intensity profile of the optical excitation and allows to modify the spin-wave spectrum observed. Despite of the ultrashort character of the excitation, the temperature profile remains over the range of our observation of one nanosecond. This change in saturation magnetization impacts the position dependent eigenfrequency supported locally and a controlled magnetic non-uniformity can be formed by the local absorption of the femtosecond laser pulse in space and time. Secondly, the laser excited spin-wave excitation results in a large spin-wave density and is leading to a high probability for scattering between spin waves and a reduced mean free path, an effect known from hot phonon localization. In addition, spin waves traveling away from the spot of excitation would propagate towards an increasing effective saturation magnetization due to the heat gradient imposed by the pump laser, so that a frequency up-conversion is needed to adopt to the local spin-wave frequency at the boundary to the cooler region. This imposes additional scattering as spin waves are continuously reflected when entering a colder region with higher saturation magnetization. Thus, an interesting scenario for a dynamic modification of magnetic film properties via femtosecond laser pulses has been demonstrated.

## Methods

To simulate the thermal response of the thin film, the heat diffusion equation


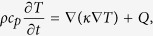


is solved in rotational symmetry for isolating sample edges and a fixed temperature at the bottom of the substrate using the material parameters listed in table 1.

Starting from equilibrium at room temperature, energy is deposited by an ultrashort laser pulse with a duration of 50 fs. The optical penetration depth is 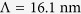 in accordance with the value for ruthenium^14^ as well as with the average value of cobalt and iron, respectively^15^. In the film plane, a Gaussian intensity profile is assumed with a FWHM of 60 m. The energy carried by the pulse amounts to a total of 1.6 *μJ* (total fluence of 13 *mJ* *cm*^−2^). Magnetization dynamics experiments were conducted on amorphous 50 nm-thick Co_40_Fe_40_B_20_ films magnetron-sputtered onto a Si(100) substrate and capped with a 3 nm Ru layer to prevent oxidation^16^.

Ultrashort laser pulses (central wavelength 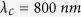, pulse duration 50 *fs*) amplified by a Coherent RegA 9040 regenerative amplifier (250 KHz repetition rate) were used to excite and detect the magnetization dynamics in a pump-probe experiment. The angle of incidence of the probe beam was 25° to the surface, while the pump beam impinged the surface perpendicularly. The pump and probe beams are focused to Gaussian spots with 93 and 24 *μm* FWHM respectively. This is mainly limiting the spatial resolution. The expected experimental width will be given by pump-and probe beam profiles convoluted. However the width of the Gaussian can be determined with a much higher precision than that of the probe-beam’s FWHM. Double-modulation technique was used to detect the time-resolved longitudinal component of magnetic precession: The pump intensity is modulated at 800 Hz by a chopper and the probe beam’s polarization is modulated at 50 kHz by a photoelastic modulator.

## Additional Information

**How to cite this article**: Busse, F. *et al.* A scenario for magnonic spin-wave traps. *Sci. Rep.* **5**, 12824; doi: 10.1038/srep12824 (2015).

## Figures and Tables

**Table 1:** Material parameters of the *COMSOL* simulation for 3 *nm* Ru/50 *nm* CoFeB/50 *μ*m Si sample: Density *ρ*, heat capacity *c*_*p*_, and thermal conductivity 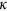 and reflectivity *R* at *λ* = 800 nm.

Material	*ρ* (kg m^−3^)	*c*_*p*_(J kg^−1^K^−1^)	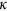 (W m^−1^K^−1^)	*R*
Ru	12370^17^	238^17^	117^17^	0.70^18^
Co_20_Fe_60_B_20_	7700^19^	440^17^	87^17^	0.72^15^
Si	2330^20^	712^20^	153^20^	–

CoFeB values *ρ* and *c*_*p*_ are average values for Co and Fe, CoFeB reflectivity is approximated by the value for Co.
